# Digital health technologies for self-identification of the risk of perinatal mental illness: a systematic review

**DOI:** 10.3389/fpubh.2026.1824057

**Published:** 2026-09-01

**Authors:** Sophie Hafner, Yui Hidaka, Jean Lillian Paul, Christine Hörtnagl, Ingrid Zechmeister-Koss

**Affiliations:** 1 Paracelsus Medical Private University, Master Programme Public Health, Center for Public Health and Healthcare Research, Salzburg, Austria; 2 Austrian Institute for Health Technology Assessment GmbH (AIHTA), Vienna, Austria; 3 Department of Psychiatry, Psychotherapy, Psychosomatics, and Medical Psychology, Division of Psychiatry I, Medical University of Innsbruck, Innsbruck, Austria; 4 Tirol Kliniken, Innsbruck, Austria

**Keywords:** digital health technologies, early detection, mental health screening, perinatal mental illness, self-identification

## Abstract

**Background:**

Perinatal mental illness affects up to 20% of mothers and 10% of fathers and is associated with adverse parental and child outcomes. Digital health technologies enabling self-identification of perinatal mental illness risk may improve early detection and reduce access barriers. This systematic review evaluated the effectiveness and safety of digital health technologies for self-identification of the risk of perinatal mental illness and examined social, organizational, and legal aspects relevant to their implementation.

**Methods:**

A systematic review with narrative synthesis was conducted in accordance with the Preferred Reporting Items for Systematic Reviews and Meta-Analyses 2020 statement and Cochrane Handbook for Systematic Reviews of Interventions. Searches were performed in PubMed, CINAHL, Web of Science, PsycINFO, and the Cochrane Library, complemented by gray literature screening and hand search. Methodological quality was assessed using established appraisal tools for the primary outcomes.

**Results:**

Of 2,762 records identified, seven publications were included. Only one prospective cohort study evaluated effectiveness, showing fair to moderate agreement between digital and conventional methods, with increased risk of bias. Safety data were limited as well. Six studies consistently reported favorable user-related outcomes. No empirical evidence addressed organizational or legal implementation aspects.

**Discussion:**

Despite consistently positive user-related findings, robust evidence on effectiveness, safety, and system-level implementation is lacking. High-quality research is required before responsible integration into routine perinatal mental healthcare can be considered.

**Systematic review registration:**

https://doi.org/10.17605/OSF.IO/H78YQ.

## 1 Introduction

Perinatal mental illness affects up to one in five mothers (1) and approximately one in ten fathers (2), representing a major public health challenge. The condition is associated with substantial personal, familial, and societal burden (3). Given its prevalence and impact across generations, improving early detection of perinatal mental illness is a key priority for public health (4).

Perinatal mental illness encompasses depression and anxiety disorders, which occur most frequently and have been extensively researched, but also psychosis and post-traumatic stress disorder (1). It is associated with adverse pregnancy outcomes (5), long-term emotional and cognitive difficulties in children, as well as impaired parent–child bonding (6). In addition, perinatal mental illness increases morbidity and mortality among parents (5) and places a substantial economic burden on healthcare systems, with most costs driven by long-term consequences for affected children (7).

Early identification is essential to reduce adverse outcomes for parents and children (4). However, access to timely screening is limited by individual barriers, including shame, stigma (8), and lack of trust (9), as well as organizational challenges (10). System-level constraints include limited financial resources (11), limited time, and absent care pathways (12). Even when perinatal mental illness is identified, care is not guaranteed, as only 15%–25% of individuals detected through screening subsequently receive treatment (13).

Digital self-identification has emerged as a promising approach in perinatal mental healthcare to contribute to early risk identification (14) and address previously described access barriers (15). Schomerus et al. (16) defined self-identification of mental illness as a dynamic cognitive process involving the assessment of current health complaints and the awareness of personal vulnerability to mental illness. In our review, self-identification refers to assessing the risk of perinatal mental illness and becoming aware of this condition by independently answering validated and reliable questionnaires without professional assistance. This can be delivered through digital health technologies, such as applications, programs and software solutions (17). In practical terms, users independently answer standardized questions on mental health symptoms or psychosocial risk and may receive feedback on possible risk and guidance towards professional assessment, support, or referral. In our review, digital self-identification is treated as a narrowly defined, user-led subset of the broader digital screening literature. It is therefore conceptually related to digital self-screening and self-assessment but does not constitute clinical diagnosis. Digital screening is a broader process for identifying individuals at increased risk and may involve professional interpretation, further assessment, and referral, whereas risk assessment may additionally incorporate clinical judgment and multiple sources of information (18). Evidence suggests that digital self-identification may support early detection (14). In the context of workforce shortages and increasing demands on healthcare systems, digital tools additionally offer opportunities for more resource-efficient care delivery and broader reach (19). Given the growing interest in digital approaches for perinatal mental health, it is important to clarify key aspects related to their clinical use and integration into routine care.

Previous evidence syntheses have examined digital technologies in perinatal mental health but have generally included heterogeneous intervention types, such as screening, education, mindfulness, self-help, and therapy support (e.g., (20, 21)).

Feldman et al. (20) reviewed the risks and benefits for women with perinatal mental illness across several intervention types, whereas Spadaro et al. (22) focused on diagnostic and screening applications identified through commercial app stores. Clarke et al. (23) examined digital mental health screening more broadly but were not limited to independent self-identification. To the best of our knowledge, no previous systematic review has focused specifically on digital self-identification of the risk of perinatal mental illness.

To address this gap the present systematic review aimed to evaluate the effectiveness and safety of digital health technologies for self-identification of the risk of perinatal mental illness. These domains were selected because digital self-identification can contribute meaningfully to early detection only if it identifies individuals at increased risk with sufficient reliability and without exposing users, healthcare professionals, or their data to avoidable harm. In this review, therefore effectiveness referred to the performance and potential benefits of the self-identification and early-detection process.

The secondary aim was to synthesize available evidence on social, organizational, and legal aspects relevant to their implementation in healthcare systems. These social aspects comprised user-related outcomes (including availability and access, acceptance and usability, user engagement, user experience) and ethical considerations.

## 2 Methods

### 2.1 Study design

The study design of a systematic review was chosen to examine specific clinically relevant topics, including effectiveness, safety, and related clinical outcomes. The review was conducted in accordance with the Preferred Reporting Items for Systematic reviews and Meta-Analyses (PRISMA) 2020 statement paper (24) and the Cochrane Handbook for Systematic Reviews of Interventions, Version 6.4 (25).

The review protocol was developed *a*.*priori* and preregistered on the Open Science Framework (OSF) on July 1, 2024 (DOI https://doi.org/10.17605/OSF.IO/H78YQ).

### 2.2 Eligibility criteria

Inclusion and exclusion criteria were predefined in the review protocol based on the Population, Intervention, Comparator, Outcome, and Study design (PICOS) framework. The eligible population included parents and parents-to-be during pregnancy and up to one year postpartum, encompassing mothers, fathers, and diverse family constellations. Included interventions were digital health technologies for the self-identification of the risk of perinatal mental illness. For eligibility purposes, digital self-identification tools were defined as digital health technologies that enabled users to independently assess their risk of perinatal mental illness by completing validated, standardized, or study-defined screening or assessment instruments without direct professional assistance. As the search strategy included broader concepts such as screening, early detection, and self-assessment, potentially relevant studies were assessed against the operational definition of digital self-identification during full-text review. Related digital screening or assessment studies were excluded when the assessment was primarily administered by a healthcare professional and did not include a user-led risk-identification component.

Studies focusing on non-digital approaches, prevention or treatment interventions, mental illness outside the perinatal period, or technology development without evaluation were excluded. Comparators included no intervention or conventional non-digital assessments.

The outcome domains were predefined based on the Health Technology Assessment (HTA) Core Model, Version 3.0, and the research questions specified in the review protocol. Primary outcomes comprised the clinical domains of effectiveness and safety. Effectiveness referred to the performance and potential benefits of the self-identification and early-detection process rather than to treatment efficacy. Predefined effectiveness outcomes included diagnostic accuracy and agreement in comparison with validated or conventional assessment methods, including sensitivity, specificity, positive predictive value, and negative predictive value. Where reported, additional effectiveness outcomes included detection rates, mental health symptoms and symptom severity, time to accurate diagnosis, and time to treatment.

Safety comprised patient safety, professional safety, data security, and privacy. Relevant outcomes included potential harms associated with false-positive or false-negative results, missed urgent cases, over-detection, inappropriate care pathways, unclear professional responsibilities or referral pathways, and secure data handling.

Secondary outcomes addressed social (including availability and access, acceptance and usability, user engagement, user experience, and ethical considerations), organizational, and legal aspects relevant to the implementation of these technologies. For effectiveness and safety outcomes, randomized controlled trials, non-randomized controlled trials, prospective cohort studies, and systematic reviews were eligible. No restrictions on study design were applied for secondary outcomes. Only studies published in English or German between 2014 and July 2024 were considered.

### 2.3 Search strategy

A systematic literature search was conducted on July 1st, 2024, using a predefined search strategy across PubMed, CINAHL, Web of Science, PsycINFO, and the Cochrane Library, as well as the electronic library of the Medical University of Vienna (see Supplementary file S1 for search strategy).

In addition, gray literature was searched, including the International Network of Agencies for Health Technology Assessment (INAHTA) and the AWMF (Arbeitsgemeinschaft der Wissenschaftlichen Medizinischen Fachgesellschaften e. V.) guideline database. Hand search of relevant journals and Google Scholar was performed, and reference lists of eligible publications were screened to identify additional studies.

### 2.4 Study selection and screening process

After automated deduplication using Deduklick, records were manually checked for remaining duplicates. Titles and abstracts were independently screened by the lead author (SH) and the second reviewer (YH) using computer-assisted and blinded procedures, following a pilot-testing phase. Full-text articles were subsequently assessed for eligibility by the same reviewers in a blinded manner, again after pilot testing. Conflicts were resolved through discussion, and a third reviewer (IZK) was consulted when consensus could not be reached.

Full-text articles excluded during eligibility assessment and their primary reasons for exclusion are presented in Supplementary Table S2.

### 2.5 Data extraction and synthesis

Data were extracted using a predefined and pilot-tested extraction form by the lead author (SH) and verified by two supervisors (YH, IZK). The form covered study characteristics, intervention components, and outcome measures. Study characteristics included design, setting, participant demographics, funding, and conflicts of interest. Intervention data comprised features of the digital self-identification tools, assessment procedures, feedback mechanisms, professional involvement, and measured outcomes. To characterize variation among the included interventions, an author-developed descriptive three-stage classification of the degree of self-identification was applied. It was based on extracted intervention characteristics relevant to the operational definition of digital self-identification, particularly user autonomy, prior explanation or training, institutional setting, availability of assistance, and provision of feedback. The classification distinguished between fully autonomous self-identification with feedback, independent completion after prior explanation or training, and institutionally situated assessment, availability of assistance, or absence of feedback. It was developed and applied by the lead author before data extraction and narrative synthesis, discussed with the supervising authors, and used for descriptive purposes only rather than as a validated scoring system or eligibility or quality criterion.

Primary outcomes encompassed effectiveness and safety, while secondary outcomes addressed social, organizational, and legal domains. A narrative synthesis approach was applied because substantial heterogeneity among the included studies was anticipated *a*.*priori*, with regard to study designs. This heterogeneity precluded quantitative meta-analysis and required a descriptive synthesis of the available evidence according to predefined outcome domains.

Where eligible reviews were identified, review-level findings were synthesized separately from primary-study findings and were not combined with primary-study data in order to avoid double counting in case of potential overlap.

### 2.6 Quality assessment

The methodological quality and risk of bias of studies contributing to the primary outcomes were assessed using predefined appraisal tools according to study design (see Table 1). Systematic reviews were evaluated using ROBIS (26), randomized controlled trials with RoB 2 (27) and non-randomized studies with ROBINS-I (28). CASP tools had initially been specified for other eligible study designs (29).

**Table 1:** Predefined quality assessment tools.

Study design	Quality assessment tool
Systematic reviews	Risk of bias in systematic reviews (ROBIS) (26)
Individually randomized parallel-group trials	Risk of bias tool 2 (RoB2) (27)
Non-randomized studies	The risk of bias in non-randomized studies - of interventions (ROBINS-I) (28)
All others- various scientific literature	Critical appraisal skills program (CASP) (29)
Refinement: eligible studies evaluating diagnostic performance	Quality assessment of diagnostic accuracy studies, version 2 (QUADAS-2) (31)

Following detailed examination of the prospective cohort study by Lawson et al. (30), QUADAS-2 was considered the most appropriate tool for evaluating its diagnostic performance component because the prospective cohort study compared a digital index assessment with a reference assessment and reported agreement and diagnostic performance measures (31). This refinement of the appraisal approach was discussed and agreed upon by the review team.

Quality assessment was performed independently by the lead author (SH) and verified by a second reviewer (YH). Any discrepancies were discussed until consensus was reached.

### 2.7 Considerations regarding study inclusion

One included study involved participants beyond 12 months postpartum; however, it was retained because the mean postnatal age at the time of digital technology use was 15.28 weeks (32).

In the study by Nurbaeti et al. (14), the postnatal period was not explicitly defined in weeks, months, or year(s). As commonly used definitions of the postnatal period extend up to 1 year after birth, inclusion was considered appropriate following reviewer discussion (33, 34).

The study by Highet et al. (35) was included despite being conducted in a hospital waiting room, as the intervention was implemented as a self-identification process. Although assistance from healthcare staff was possible, no such cases were reported in the results, and inclusion was therefore considered acceptable (35).

## 3 Results

**Figure 1:**
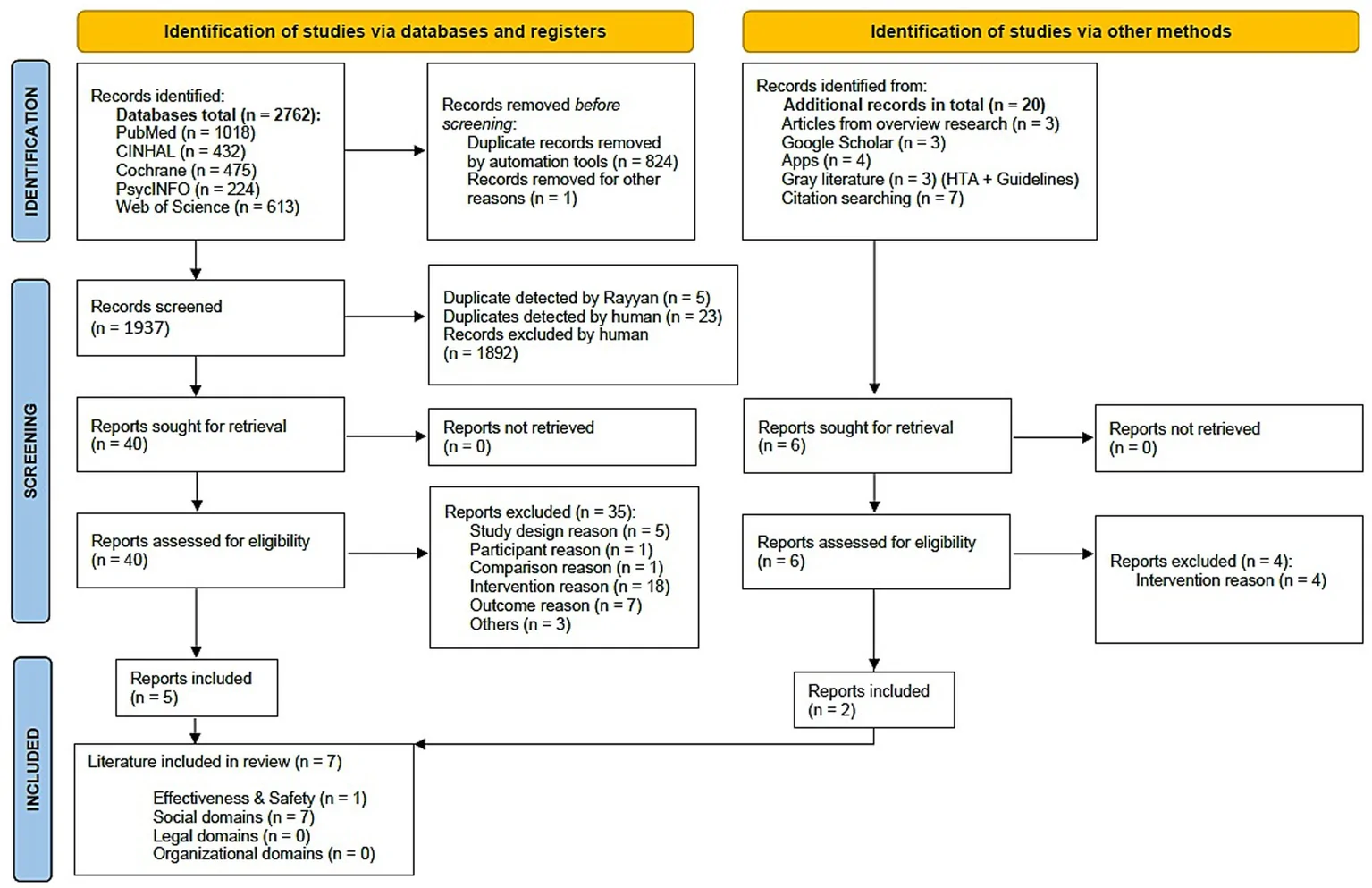
PRISMA flow diagram (based on (49)).

The study selection process, illustrated in the PRISMA flow diagram (see Figure 1), initially identified 2,762 records, of which 852 duplicates were removed. After title and abstract screening, 40 records were considered potentially eligible. An additional six records were identified through manual searching, resulting in 46 full-text articles assessed for eligibility. Ultimately, six studies and one review met the inclusion criteria. The six primary studies formed empirical evidence base for effectiveness, safety, and user-related outcomes. The included review was synthesized separately and contributed only to ethical considerations within the social domain.

### 3.1 Study characteristics and components

The included papers were published between 2019 and 2024. The six studies were conducted in Germany, the United Kingdom, Canada, Indonesia, and Australia (14, 30, 32, 35–37). Study designs comprised mixed-methods (*n* = 2) (36, 37), cross-sectional (*n* = 2) (14, 32), descriptive (*n* = 1) (35), and prospective cohort studies (*n* = 1) (30), and one review (*n* = 1) (38). Sample sizes varied from 15 to 937 participants.

All studies (*n* = 6) included postnatal women, while two also involved pregnant women (32, 37). Additionally, one study included partners (37), and one study involved healthcare professionals as a stakeholder group and reported their perspectives on acceptance and implementation-related aspects separately (36).

The digital interventions were heterogeneous in target condition, delivery mode, technological format, and level of self-identification. The majority primarily addressed postnatal depression (14, 30, 36, 37), with some additionally targeting antenatal depression (32) or psychosocial risk (32, 35). Five interventions were delivered remotely (14, 30, 32, 36, 37) and one was conducted in a clinic waiting room (35). Digital health tools included mobile applications, a web-based application and tool, a web platform, and a text messaging intervention. The degree of self-identification ranged from fully autonomous use to institutionally initiated assessments with professional involvement. Validated screening instruments for assessing the mental health status were commonly used, most frequently the Edinburgh Postnatal Depression Scale (EPDS) (*n* = 4), with varying cutoff scores and feedback mechanisms (14, 35–37). An overview of the study characteristics is provided in Table 2. The full data extraction table is presented in Supplementary Table S1.

**Table 2:** Characteristics of included studies.

Study (year)	Country	Study design	Sample size	Population	Digital format	Target condition	Instrument
Lawson et al. (2019) (30)	Australia	Prospective cohort	937	Postnatal women	Text messaging	Postnatal depression	PHQ-2
Eisner et al. (2022) (37)	United Kingdom	Mixed-methods	15 + 8	Pregnant & postnatal women (+ partners)	Mobile app	Postnatal depression	EPDS
Daehn et al. (2023) (36)	Germany	Mixed-methods	217	Postnatal women (+ professionals)	Web app	Postnatal depression	EPDS
Highet et al. (2019) (35)	Australia	Descriptive cohort	144	Postnatal women	Web-based tool	Postnatal depression + psychosocial risk	EPDS + Self-composed questions
Nurbaeti et al. (2021) (14)	Indonesia	Cross-sectional	109	Postnatal women	Mobile app	Postnatal depression	EPDS
Reilly and Austin (2021) (32)	Canada	Cross-sectional	140	Pregnant & postnatal women	Web app	perinatal depression + psychosocial risk	The Whooley questions + ANQR
Fonseca et al. (2024) (38)	–	Review	–	–	–	Ethical aspects	

### 3.2 Synthesis of findings

#### 3.2.1 Primary outcomes

##### 3.2.1.1 Effectiveness

One study met the eligibility criteria and evaluated the effectiveness of digital health technologies for self-identification of the risk of perinatal mental illness (30). It compared a biweekly text message assessment based on the Patient Health Questionnaire-2 (PHQ-2) for identifying the risk of postnatal depression with EPDS administered via telephone interview as the reference instrument. Agreement between the two instruments was fair (Cohen’s *κ* = 0.37) using the initial cutoff and moderate (*κ* = 0.45) after adjustment of the cutoff score. The balance of sensitivity, specificity, positive predictive value, and negative predictive value varied depending on the applied cutoff (30). No other included study reported effectiveness outcomes as predefined in the review protocol.

##### 3.2.1.2 Safety

Regarding safety, limited data were reported in the same study, primarily concerning false-positive screening results and subsequent follow-up outcomes (see Supplementary Figure S1) (30). No further data on patient safety, professional safety, or data security were reported in any of the included studies.

#### 3.2.2 Secondary outcomes

##### 3.2.2.1 Social domain

Six studies examined secondary social outcomes, including user-related and ethical aspects, across the included digital health technologies (14, 30, 32, 35–37). Overall, availability and access varied across studies regarding platform type, geographic reach, language options, and target population. None of the studies reported regulatory approval or certification status (e.g., certification as a medical device).

Acceptance and usability were assessed using validated instruments or study-specific questionnaires and were consistently rated as high across studies (14, 30, 32, 35–37). User engagement measures showed high completion rates (30, 35–37), although declining participation was observed in repeated assessments (30, 37). User experiences were generally positive (14, 32), and one study also reported favorable feedback from health professionals (36).

Ethical aspects were addressed in only one non-clinical review (38), highlighting key concerns related to autonomy, privacy, data security, and equity in the use of digital self-identification tools to identify the risk of perinatal mental illness. The review further described potential benefits related to improved access and flexible risk assessment, as well as concerns regarding regulatory oversight and the robustness of the evidence base (38).

##### 3.2.2.2 Organizational and legal domain

No empirical evidence was identified on organizational or legal aspects related to implementing digital self-identification tools in perinatal mental health routine care. National guidelines on prevention and early detection exist, but they do not address digital self-identification. In addition, none of the included studies examined regulation, licensing, or liability.

### 3.3 Risk of bias

As only one study could be included for the primary outcomes according to the inclusion and exclusion criteria, the study by Lawson et al. (30) was critically appraised. They used a prospective cohort design and evaluated the screening performance of the digital assessment by comparing the text-message-based PHQ-2 with the EPDS as a reference assessment (31). As this evaluation involved an index assessment, a reference assessment, and measures of agreement and diagnostic performance, QUADAS-2 was applied to assess the risk of bias and applicability of this component. An increased risk of bias in three out of four domains could be identified (Table 3). The results of the study by Lawson et al. (30) should consequently be interpreted in light of these limitations.

**Table 3:** Risk of bias according to QUADAS-2 (31) - evaluation for Lawson et al. (30) study.

Study	Risk of bias				Applicability concerns		
	Patient selection	Index test	Reference standard	Flow and timing	Patient selection	Index test	Reference standard
Study 1	☹	☺	☹	☹	☺	☺	☺

☺ Low risk, ☹ High risk, ? Unclear risk.

## 4 Discussion

This systematic review revealed substantial gaps in evidence regarding the effectiveness, safety, and healthcare system implementation of digital health technologies for self-identification of the risk of perinatal mental illness, despite consistently high user-related findings. Only one prospective cohort study evaluated effectiveness and found fair to moderate agreement between digital self-identification and conventional assessment methods. Safety data were scarce and were assessed exclusively in this study, being limited to false-positive follow-up data. In contrast, six included studies examined social aspects. They primarily focused on user-related outcomes such as acceptance and usability, user engagement, and user experience. Across different digital formats, these outcomes were consistently rated as positive. Ethical considerations within these social aspects were addressed in one non-clinical review. This review emphasized improved accessibility, while highlighting potential risks related to autonomy, privacy, and equity. No included studies examined organizational or legal aspects relevant to the implementation in routine perinatal mental healthcare.

The current evidence base does not allow firm conclusions to be drawn regarding the effectiveness and safety of digital health technologies for self-identification of the risk of perinatal mental illness. Based on a single prospective cohort study, the findings cannot be generalized and should be interpreted as preliminary and hypothesis-generating only. Moreover, the available literature was heterogeneous with regard to study design, intervention and individual inclusion criteria, which further limits comparability across studies. The small number of included publications should therefore be interpreted in light of both the current stage of research in this field and the operational definition applied in this review. It does not necessarily indicate an absence of digital screening or assessment research in perinatal mental health more broadly but reflects the narrower focus on user-led digital self-identification as distinct from professionally administered screening, prevention, treatment, or general digital mental health interventions.

Regarding study characteristics, almost all included studies focused on women, indicating that men’s mental health during the antenatal and postnatal periods remains underrepresented in this evidence base. This pattern is consistent with previous literature showing that paternal perinatal mental health remains less frequently addressed despite specific barriers to help-seeking, including stigma and limited awareness of paternal mental health needs (39). Future research should therefore examine digital self-identification tools specifically among fathers, partners, and more diverse family constellations.

Overall central finding of this review was not evidence of established effectiveness or safety, but the limited availability of high-quality evidence on the effectiveness of such digital formats, consistent with previous studies (21, 22). This result aligns with reviews reporting inconclusive evidence on whether screening approaches deliver more benefit than harm (40, 41). The available evidence on effectiveness was limited to the performance of the identification process, specifically agreement and diagnostic performance measures.

This narrow evidence base is particularly important when digital self-identification is considered an early-recognition strategy. Identification of an increased risk represents only an initial step and does not itself establish a diagnosis or ensure improved health outcomes. The potential benefit of screening depends on subsequent confirmation, timely professional assessment, appropriate referral, and access to effective care (42). However, the included studies did not establish whether digital self-identification leads to earlier diagnosis, increased help-seeking, improved referral or treatment uptake, or better longer-term outcomes. Digital self-identification should therefore currently be considered a potential component of a broader screening and care pathway rather than an established stand-alone early detection strategy.

Beyond the limited evidence on safety in general, concerns have also been raised regarding data protection standards of digital health technologies. Previous research not included in this review has identified security deficiencies in screening apps, including unclear information on duty of care and a lack of references to compliance with data protection laws (22). Within the European Union (EU), digital health technologies used for diagnostic purposes may fall under the General Data Protection Regulation (Regulation (EU) 2016/679). It establishes binding requirements for the processing and protection of personal data (43).

While evidence on effectiveness and safety was limited, social user-related outcomes were reported consistently and favorably. Across six included studies, positive results were reported for acceptance, usability, user engagement, and user experience, although the interventions varied substantially in design and format. Despite this heterogeneity, the overall pattern indicates high acceptance of digital self-identification tools, consistent with previous research on digital mental health screening (21, 23, 44, 45). Preferences for digital modalities suggest potential to reduce common barriers to help seeking during the perinatal period (30).

Organizational and legal aspects of digital health technologies for self-identification of the risk of perinatal mental illness remain insufficiently addressed. This research gap has important implications for implementation and regulatory oversight. It contributes to ongoing regulatory uncertainty regarding the classification of these tools, as reflected in policy and regulatory frameworks not included in this review. Within the EU, diagnostic software may be classified as a medical device, whereas lifestyle or well-being applications fall outside medical device regulation. This distinction is particularly relevant for digital self-identification tools operating at the interface between risk awareness and diagnostic support (46). Despite guidance from international frameworks such as the World Health Organization (WHO) Classification of Digital Health Interventions v 1.0 (47) and the National Institute for Health and Care Excellence (NICE) Evidence Standards Framework (48), empirical evidence to support consistent classification and regulation in perinatal mental health settings remains limited.

### 4.1 Strengths and limitations

This review has several strengths. It followed established methodological standards, including the PRISMA 2020 statement and a pre-registered protocol, thereby enhancing transparency and reproducibility. In addition, a comprehensive search strategy across multiple databases, supplemented by hand searching and gray literature, helped reduce the risk of publication bias.

This review has three main limitations. First, no widely accepted definition of “self-identification” in the context of perinatal mental health could be identified. Although an *a*.*priori* working definition was developed following an extensive literature review, the conceptual boundaries between self-identification, self-assessment, self-screening and broader risk assessment and screening remained partly ambiguous, requiring interpretative judgment during study selection.

Second, the included studies predominantly focused on women in the postnatal period, with fathers and antenatal populations markedly underrepresented. Although the eligibility criteria were intentionally broad and included pregnant individuals, postnatal individuals, and partners, the available evidence did not reflect this full target population. Moreover, most interventions addressed postnatal depression, while other relevant conditions such as anxiety disorders, post-traumatic stress disorders, and mental disorders during pregnancy were rarely examined, limiting the generalizability of the findings.

Third, the evidence base was characterized by substantial heterogeneity in study designs, interventions, and inclusion criteria, alongside limited methodological quality. This heterogeneity limited comparability across studies and precluded quantitative synthesis. None of the included studies employed randomized designs, which further restricts the strength of conclusions that can be drawn from the available evidence.

### 4.2 Conclusion and further implications

The current evidence does not allow conclusions regarding the effectiveness or safety of digital health technologies for self-identification of the risk of perinatal mental illness. It further does not support routine implementation within clinical or public health practice. Despite favorable user-related outcomes, robust evidence on effectiveness and safety is lacking. Furthermore, empirical data on organizational and legal requirements for implementation in healthcare systems are largely absent. These gaps substantially limit implementation readiness.

Digital self-identification tools may support earlier risk recognition if they are embedded within clearly defined care pathways and supported by appropriate governance structures. However, implementation should only be considered once sufficient evidence on effectiveness, safety, and organizational and legal frameworks is available. Establishing high-quality evidence in these domains is essential before responsible and evidence-based integration into routine perinatal mental healthcare can be recommended.

## Data Availability

The original contributions presented in the study are included in the article/Supplementary material, further inquiries can be directed to the corresponding author.
