# Supplementary material for: Digital health technologies for self-identification of the risk of perinatal mental illness: a systematic review
Source: Front Public Health. 2026 Sep 1;14:1824057. doi: 10.3389/fpubh.2026.1824057 (PMC13574571; doi:10.3389/fpubh.2026.1824057)
Supplement: Supplementary file 1 [file Supplementary_file_1.pdf]

Supplementary Material

1 Supplementary File

Supplementary File S1. Full electronic search strategy for PubMed.

#1	Population	parent* OR mother* OR mom OR mum OR father* OR dad OR co-parent* OR (adoptive parent*) OR parent*-to-be OR (expect* parent*) OR "mother*-to-be" OR "father*-to-be" OR pregnant OR pregnancy
#2	Digital health technology	“digital technolog*” OR “digital health technolog*” OR digital OR online OR mobile OR (mobile AND (phone OR application OR technolog*)) OR “smartphone technolog*” OR platform OR website OR mHealth OR eHealth OR telemedicine OR telehealth OR (internet-delivered program*) OR telepsychiatry OR “remote sensing” OR computer OR software OR ((web OR computer OR internet OR online) AND (based OR assisted OR guided OR aided OR delivered OR supported))
#3	Self-identification	screening OR “early detection” OR “self-assessment” OR (self-identification)
#4	Perinatal mental illness	(perinatal OR peripartum OR peripartal OR prenatal OR antenatal OR postnatal OR postpartum) AND (((mental OR psychological) AND (health OR illness OR disease OR disorder OR condition)) OR “mood disorder*” OR (depress* OR psychosis OR anxiety OR (post traumatic stress disorder)))

01.07.2024

total hits 1,018

09:56:47/ Austrian Time 15:56, 01.07.2024

Search: #1 AND #2 AND #3 AND #4 Filters: from 2014 - 2024

#1 (("parent*"[All Fields] OR "mother*"[All Fields] OR "mom"[All Fields] OR ("mum int conf mob ubiquitous multimed"[Journal] OR "mum"[All Fields]) OR "father*"[All Fields] OR "dad"[All Fields] OR "co parent*"[All Fields] OR (("adoptive"[All Fields] OR "adoptively"[All Fields]) AND "parent*"[All Fields]) OR "parent* to be"[All Fields] OR ("expect*"[All Fields] AND "parent*"[All Fields]) OR "mother* to be"[All Fields] OR "father* to be"[All Fields] OR ("pregnant"[All Fields]

OR "pregnants"[All Fields]) OR ("pregnancy"[MeSH Terms] OR "pregnancy"[All Fields] OR "pregnancies"[All Fields] OR "pregnancy s"[All Fields]))

#2 AND ("digital technolog*"[All Fields] OR "digital health technolog*"[All Fields] OR ("digitalisation"[All Fields] OR "digitalised"[All Fields] OR "digitalization"[All Fields] OR "digitalize"[All Fields] OR "digitalized"[All Fields] OR "digitalizer"[All Fields] OR "digitalizing"[All Fields] OR "digitally"[All Fields] OR "digitals"[All Fields] OR "digitization"[All Fields] OR "digitizations"[All Fields] OR "digitize"[All Fields] OR "digitized"[All Fields] OR "digitizer"[All Fields] OR "digitizers"[All Fields] OR "digitizes"[All Fields] OR "digitizing"[All Fields] OR "radiographic image enhancement"[MeSH Terms] OR ("radiographic"[All Fields] AND "image"[All Fields] AND "enhancement"[All Fields]) OR "radiographic image enhancement"[All Fields] OR "digital"[All Fields]) OR "online"[All Fields] OR ("mobile"[All Fields] OR "mobiles"[All Fields]) OR (("mobile"[All Fields] OR "mobiles"[All Fields]) AND ("phone s"[All Fields] OR "phoned"[All Fields] OR "phones"[All Fields] OR "phoning"[All Fields] OR "telephone"[MeSH Terms] OR "telephone"[All Fields] OR "phone"[All Fields] OR ("applicabilities"[All Fields] OR "applicability"[All Fields] OR "application"[All Fields] OR "applications"[All Fields] OR "applicative"[All Fields]) OR "technolog*"[All Fields])) OR "smartphone technolog*"[All Fields] OR ("platform"[All Fields] OR "platform s"[All Fields] OR "platforms"[All Fields]) OR ("website"[All Fields] OR "website s"[All Fields] OR "websites"[All Fields]) OR ("mhealth s"[All Fields] OR "telemedicine"[MeSH Terms] OR "telemedicine"[All Fields] OR "mhealth"[All Fields]) OR ("telemedicine"[MeSH Terms] OR "telemedicine"[All Fields] OR "ehealth"[All Fields]) OR ("telemedicine"[MeSH Terms] OR "telemedicine"[All Fields] OR "telemedicine s"[All Fields]) OR ("telehealth s"[All Fields] OR "telemedicine"[MeSH Terms] OR "telemedicine"[All Fields] OR "telehealth"[All Fields]) OR ("internet-delivered"[All Fields] AND "program*"[All Fields]) OR "telepsychiatry"[All Fields] OR "remote sensing"[All Fields] OR ("computability"[All Fields] OR "computable"[All Fields] OR "computating"[All Fields] OR "computation"[All Fields] OR "computational"[All Fields] OR "computations"[All Fields] OR "compute"[All Fields] OR "computed"[All Fields] OR "computer s"[All Fields] OR "computers"[MeSH Terms] OR "computers"[All Fields] OR "computer"[All Fields] OR "computes"[All Fields] OR "computing"[All Fields] OR "computional"[All Fields]) OR ("software"[MeSH Terms] OR "software"[All Fields] OR "software s"[All Fields] OR "softwares"[All Fields]) OR (("web"[All Fields] OR ("computability"[All Fields] OR "computable"[All Fields] OR "computating"[All Fields] OR "computation"[All Fields] OR "computational"[All Fields] OR "computations"[All Fields] OR "compute"[All Fields] OR "computed"[All Fields] OR "computer s"[All Fields] OR "computers"[MeSH Terms] OR "computers"[All Fields] OR "computer"[All Fields] OR "computes"[All Fields] OR "computing"[All Fields] OR "computional"[All Fields]) OR ("internet"[MeSH Terms] OR "internet"[All Fields] OR "internet s"[All Fields] OR "internets"[All Fields]) OR "online"[All Fields]) AND ("based"[All Fields] OR "basing"[All Fields] OR ("assistances"[All Fields] OR "assistant s"[All Fields] OR "assistants"[All Fields] OR "assisted"[All Fields] OR "assisting"[All Fields] OR "assistive"[All Fields] OR "dental assistants"[MeSH Terms] OR ("dental"[All Fields] AND "assistants"[All Fields]) OR "dental assistants"[All Fields] OR "assistant"[All Fields] OR "helping behavior"[MeSH Terms] OR ("helping"[All Fields] AND "behavior"[All Fields]) OR "helping behavior"[All Fields] OR "assist"[All Fields] OR "assistance"[All Fields] OR "assists"[All Fields]) OR ("guide"[All Fields] OR "guided"[All Fields] OR "guides"[All Fields] OR "guiding"[All Fields]) OR ("aided"[All Fields] OR "aiding"[All Fields]) OR ("deliver"[All Fields] OR "delivered"[All Fields] OR "delivering"[All Fields] OR "delivers"[All Fields]) OR ("support"[All Fields] OR "support s"[All Fields] OR

"supported"[All Fields] OR "supporter"[All Fields] OR "supporter s"[All Fields] OR "supporters"[All Fields] OR "supporting"[All Fields] OR "supportive"[All Fields] OR "supportiveness"[All Fields] OR "supports"[All Fields]))))

#3 AND ("diagnosis"[MeSH Subheading] OR "diagnosis"[All Fields] OR "screening"[All Fields] OR "mass screening"[MeSH Terms] OR ("mass"[All Fields] AND "screening"[All Fields]) OR "mass screening"[All Fields] OR "early detection of cancer"[MeSH Terms] OR ("early"[All Fields] AND "detection"[All Fields] AND "cancer"[All Fields]) OR "early detection of cancer"[All Fields] OR "screen"[All Fields] OR "screenings"[All Fields] OR "screened"[All Fields] OR "screens"[All Fields] OR "early detection"[All Fields] OR "self-assessment"[All Fields] OR "self-identification"[All Fields]) AND (("perinatal"[All Fields] OR "perinatally"[All Fields] OR "perinatals"[All Fields] OR ("peripartum period"[MeSH Terms] OR ("peripartum"[All Fields] AND "period"[All Fields]) OR "peripartum period"[All Fields] OR "peripartum"[All Fields]) OR "peripartal"[All Fields] OR ("prenatal"[All Fields] OR "prenatally"[All Fields] OR "prenatals"[All Fields]) OR ("antenatal"[All Fields] OR "antenatally"[All Fields]) OR ("postnatal"[All Fields] OR "postnatally"[All Fields]) OR ("postpartum period"[MeSH Terms] OR ("postpartum"[All Fields] AND "period"[All Fields]) OR "postpartum period"[All Fields] OR "postpartum"[All Fields]))

#4 AND ((("mental"[All Fields] OR "mentalities"[All Fields] OR "mentality"[All Fields] OR "mentalization"[MeSH Terms] OR "mentalization"[All Fields] OR "mentalizing"[All Fields] OR "mentalize"[All Fields] OR "mentalized"[All Fields] OR "mentally"[All Fields] OR ("psychologic"[All Fields] OR "psychological"[All Fields] OR "psychologically"[All Fields] OR "psychologization"[All Fields] OR "psychologized"[All Fields] OR "psychologizing"[All Fields])) AND ("health"[MeSH Terms] OR "health"[All Fields] OR "health s"[All Fields] OR "healthful"[All Fields] OR "healthfulness"[All Fields] OR "healths"[All Fields] OR ("illness"[All Fields] OR "illness s"[All Fields] OR "illnesses"[All Fields]) OR ("disease"[MeSH Terms] OR "disease"[All Fields] OR "diseases"[All Fields] OR "disease s"[All Fields] OR "diseased"[All Fields]) OR ("disease"[MeSH Terms] OR "disease"[All Fields] OR "disorder"[All Fields] OR "disorders"[All Fields] OR "disorder s"[All Fields] OR "disordes"[All Fields]) OR ("condition"[All Fields] OR "condition s"[All Fields] OR "conditions"[All Fields]))) OR "mood disorder*"[All Fields] OR ("depress*"[All Fields] OR ("psychotic disorders"[MeSH Terms] OR ("psychotic"[All Fields] AND "disorders"[All Fields]) OR "psychotic disorders"[All Fields] OR "psychosis"[All Fields]) OR ("anxiety"[MeSH Terms] OR "anxiety"[All Fields] OR "anxieties"[All Fields] OR "anxiety s"[All Fields]) OR ("stress disorders, post traumatic"[MeSH Terms] OR ("stress"[All Fields] AND "disorders"[All Fields] AND "post traumatic"[All Fields]) OR "post-traumatic stress disorders"[All Fields] OR ("post"[All Fields] AND "traumatic"[All Fields] AND "stress"[All Fields] AND "disorder"[All Fields]) OR "post traumatic stress disorder"[All Fields])))))

#Filter AND (2014:2024[pdat])

2 Supplementary Figures and Tables

2.1 Supplementary Figures

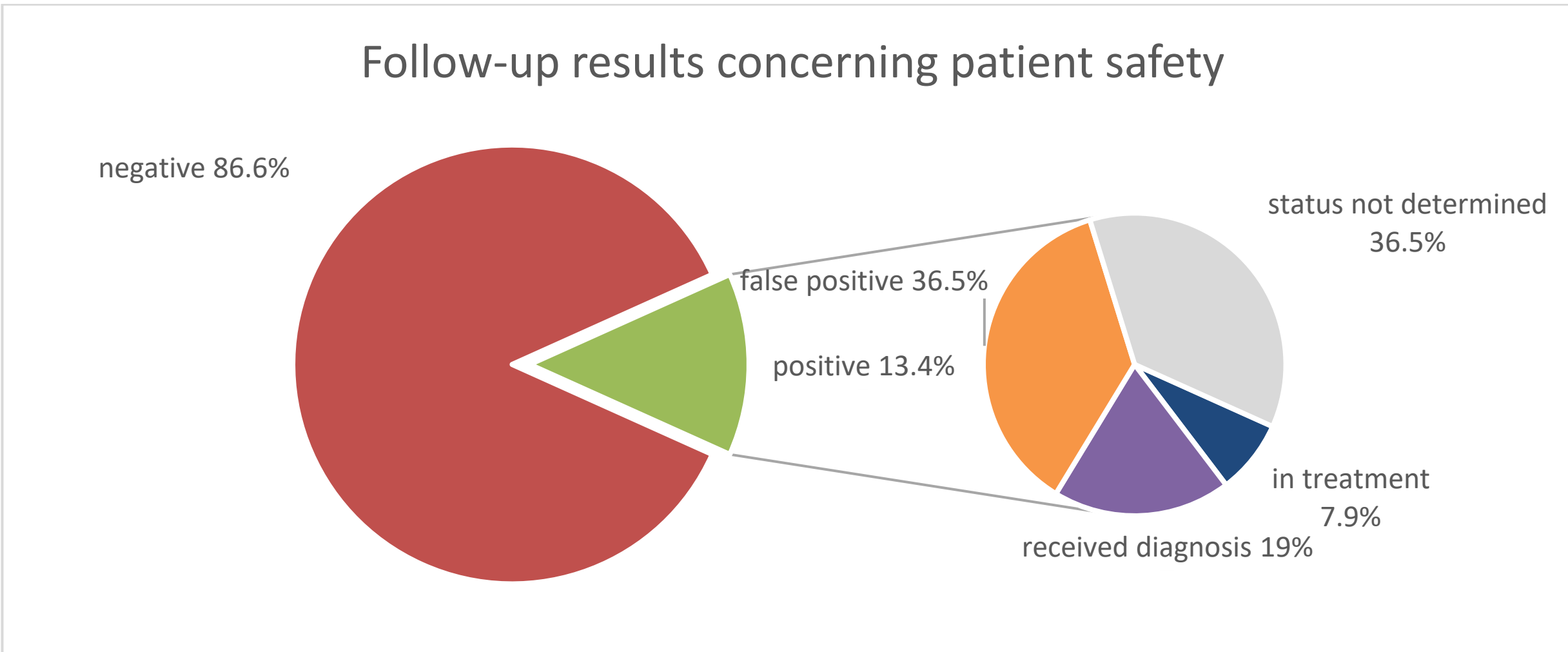

Supplementary Figure S1. Follow-up results concerning patient safety- Data from Lawson *et al.* (2019).

2.2 Supplementary Tables

Supplementary Table S1. Data extraction table.

Characteristics of the study included for primary outcomes

Legend for all tables: n.r. = data was not researched

n.a. = data was not available

Authors, Year (citation)	Lawson *et al.* (2019)
**Study characteristics**	
Country	Canada
**Study objective**	Evaluation of feasibilty of an intervention for screening for PND and providing information through text messages.
**Method**	
Study design	not specified. Seems to be prospective cohort design.
Setting of recruitment	hospital
Setting for intervention	remotely: telephone and text-messages
Setting for results follow- up	remotely: online
Study period / duration	July 2015 - January 2017 (18 months)
Follow-up period	12 to 13 weeks postpartum
Providers: (involved health care professionals or researcher)	researchers
Developer of tool	not specified- authors of the study.
Funding	Funding by the Academic Health Science Centre (AHSC) Alternative Funding Program
Conflict of interest	No financial relationships with commercial interests.
**Participants**	
No of participants	937 women postnatal
Age / mean in years (SD / range)	33.7 (SD 4.17 / range n.a.)
Weeks of pregnancy / postnatal	12 weeks postnatal
Inclusion / exclusion criteria	not specified- inclusion mentioned:<br>≥ 18 years, delivery at Mount Sinai Hospital in Toronto,<br>no language barrier (supplement)<br>+ informed consent.<br>exclusion after baseline process: women in active psychiatric care.

Components of the study included for primary outcomes

Authors, Year (citation)	Lawson *et al.* (2019)
**Study intervention**	1) baseline assessment (sociodemographic questions + EPDS)<br>2) screening by text messages (PHQ-2) biweekly until 12 weeks postpartum + informal texts 3 times / week<br>3) positiv PHQ-2 (+ matched negative PHQ-2 screened women) complete EPDS via telephone<br>4) 12-13 weeks postnatal follow-up via e-mail: satisfaction online survey (anonymous)
Features of the digital self-assessment tool	package of different interventions, not a tool.
Stage of self-identification / feedback	self-identification (3): receiving external text messages.<br>no feedback to user.
Information about further help after self-assessment.	partly: positive tested women (EPDS or PHQ-2) were offered an appointment with a perinatal psychiatrist.
Emergency Information	n.a.
Recommended pathway	n.a.
Involvement of professionals	Appointment with a perinatal psychiatrist was offered when tested positive.
**Comparator**	as a part of the study: Women, who were screened negative in phase 2 (PHQ-2).
**Outcome** parameters analyzed	evaluate the feasibility of using text messages by:<br>• agreement PHQ-2 text message and EPDS (sensitivity & specificity)<br>• user engagement<br>• user satisfaction
perinatal mental illness type	postnatal depression
Tools for assessing mental health state	EPDS (with cutoff ≥ 15 in pregnancy /baseline and ≥ 12 postpartum), PHQ-2
Additional tools used for the study	none

Results of the study included for primary outcomes

Authors, Year (citation)	Lawson *et al.* (2019)
**Results effectiveness**	
Diagnostic accuracy (sensitivity, specificity, positive/negative predictive value)	Fair agreement between text message screening and EPDS (kappa value = 0.37) / cut-off adation: 0.45<br>sensitivity: 0.49 (95 % CI = 0.38-0.61) / adaption: 0.90 (95 % CI = 0.81-0.96)<br>specificity: 0.93 (95 % CI = 0.91-0.94) / adpation: 0.82 (95 % CI = 0.79-0.85)<br>positive predictive value: 0.38 (95% CI = 0.31- 0.46) / adaption: 0.32 (95 % CI = 0.29-0.36)<br>negative predictive value: 0.95 (95 % CI = 0.94- 0.96) / adaption 0.99 (95 % CI = 0.98-0.99)
Consistency with the clinical diagnosis	Of 126 women positive screened women: 10 were already in psychological care, 10 received the diagnosis of postnatal depression, 11 postnatal psychiatric disorder diagnosis and 3 had significant psychiatric symptoms, no further data: 46.
Mental health symptoms	n.r.
Time to accurate diagnosis	n.r.
Symptom severity	n.r.
Time to treatment	n.r.
Other Benefits	n.r.
**Results safety**	
**Safety for patients**	n.r.
False-negative or false- positive results	Of 126 positive screened 34: mental illness diagnosis, 46: false-positive. 46: not diagnosed, could not be contacted or did not turn up for their appointment.
Missing or overdetection of urgent or emergency cases	n.r.
Inappropriate care pathways	n.r.
Complication rate	n.r.
Harms (e.g. psychological)	n.r.
**Data protection**	
Safety of data	Inclusion criteria was to give informed consent.
Privacy policies	n.a.
**Safety for professionals**	n.r.

Characteristics of the studies included for secondary outcomes

**Authors, Year (citation)**	**Daehn *et al.* (2023)**	**Eisner *et al.* (2022)**	**Nurbaeti *et al.* (2021)**
**Study characteristics**			
Country	Germany	United Kingdom	Indonesia
**Study objective**	Evaluation of feasibilty of web app SmartMoms, and user's and health professional's experiences and the user behaviour.	Evaluation of the smartphone app ClinTouch DAWN-P, concerning feasibiity, acceptability & usability, usage patterns, safety and the validity of app based PND screening compared to paper-based procedure.	Development and implementation of an app by using the ADDIE Model and evaluation of effectiveness.
**Method**			
Study design	mixed-methods study	mixed- methods proof-of-concept study	descriptive cross- sectional design
Setting of recruitment	healthcare facilities and providers, online (social media)	1) women: hospital + online (social media). 2) partners: via pregnant participants.	hospital
Setting for intervention	remotely: application (online), telephone.	remotely: combination of telephone calls, SMS messages, email and post.	remotely, during a homevisit through a researcher
Setting for results follow- up	no follow- up	no follow- up	no follow- up
Study period / duration	May 2021- November 2021 (6 months)	n.a.	second week of August - the second week of October 2019 (2 months)
Follow-up period	no follow-up period	no follow-up period	no follow-up
Providers (involved health care professionals or researcher)	self-initiated	self-initiated	n.a.
Developer of tool	Scientists and psychologists of University clinic Hamburg Eppendorf and Freie Universität Berlin in cooperation with external web app developers (including the authors).	not specified - authors of the study.	research team under the name of Nurbaeti Irma.
Funding	Funding is supported by a grant from the Damp Stiftung, Hamburg.	Funding by the UK Medical Research Council and Health Innovation Manchester (Mental Health and Women and Children research domains).	Funding by Universitas Islam Negeri Syarif Hidayatullah Jakarta.
Conflict of interest	No conflict of interest.	Two of the authors are directors of Affigo CIC, a not-for-profit community interest. This is a company designed to make digital health products available in the NHS and public sector.	No conflict of interest.
**Participants**			
No of participants	total: 217 women postnatal<br>usability evaluation: 129 women<br>user satisfaction and acceptability: 133 women<br>user behavior: 1874 women<br>self- screening with EPDS: 222 women<br>health care provider interviews: 13 providers	15 women, 8 partners antenatal + postnatal	109 women postnatal
Age / mean in years (SD / range)	total women: 32.5 (SD 4.8 / range 20-55)<br>EPDS maternal age: 32.7 (SD 4.0 / range 22-42)<br>health care provider 41.6 (SD 9.4 / range 27-50)	33.8 (SD 5.2 / range n.a.)	30.98 (SD n.a / range 16-50)
Weeks of pregnancy/ postnatal	12 months postnatal: average child age (in EPDS self-identification participants): 3.56 months (SD 3.7) range 0-12months	≥36 weeks pregnancy - 6 weeks postnatal	n.a.
Inclusion /exclusion criteria	not specified. inclusion mentioned: informed consent. Exclusion mentioned concerning self-screening participants: women with children over 12 months of age, pregnant women	inclusion for women:<br>≥ 36 weeks' gestation, over 18 years age, fluent in English, under the care of Manchester University NHS Foundation Trust + informed consent.<br>exclusion: current stillbirth, fetal abnormality, or multiple pregnancy<br><br>partners inclusion: male/female partner of a pregnant participant, over 18 years age, fluent in English + informed consent.	inclusion:<br>birth of a living child, married, no history of mental illness, no treatment for complications, can read Bahasa Indonesian language, smarphone<br>+ agreement

Authors, Year (citation)	Reilly & Austin (2021)	Highet *et al.* (2019)
**Study characteristics**		
Country	Australia	Australia
**Study objective**	Evaluation of the web-based tool *Mummatters c* oncerning its acceptability, risk profile of users, credibility, perceived effect and motivational appeal, and help seeking behaviors and barriers.	Evaluation of the digital screening platform iCOPE.
**Method**		
Study design	cross- sectional design	descriptive cohort design
Setting of recruitment	via web-based tool M*ummatters*	hospital
Setting for intervention	remotely: online	hospital
Setting for results follow- up	remotely: web-based Key Survey (TM) platform.	no follow- up
Study period / duration	November 13, 2016 - May 22, 2018 (18 months)	September 2015 - September 2016 (12 months)
Follow-up period	1 month follow-up period	No follow-up
Providers (involved health care professionals or researcher)	self-initiated	health professional in hospital
Developer of tool	Bupa Australia	Centre of Perinatal Excellence (COPE) and Diginostic (Diginostic Pty Ltd, 2024)
Funding	By the University of Newcastle (2018-2020), and the University of Wollongong (2020-2023)	n.a.
Conflict of interest	No conflict of interest.	n.a.
**Participants**		
No of participants	140 women: 73 antenatal and 67 postnatal	144 women postnatal
Age / mean in years (SD / range)	antenatal: 32.97 (SD 4.60 / range 24-43)<br>postnatal: 32.70 (SD 4.20 / range 25-45)	30.68 (SD 4.69 / range n.a.)
Weeks of pregnancy / postnatal	antenatal: mean 20.96 (SD 11.19), range 4-40 weeks<br>postnatal: mean 15.28 (SD 24.11), range 1-178 weeks	4-6 weeks postnatal
Inclusion / exclusion criteria	inclusion:<br>living in Australia, internet access, be able to complete the assessment in English<br>+ agreement in participation	inclusion:<br>women attending the clinic for their four to six weeks postnatal check-up<br>+ consent in usage of data

Components of the studies included for secondary outcomes

**Authors, Year (citation)**	**Daehn *et al.* (2023)**	**Eisner *et al.* (2022)**	**Nurbaeti *et al.* (2021)**
**Study intervention**	1) women: quantitative 27 item app survey (sociodemographic characteristics, app usability (SUS) and satisfaction (CSQ)) + additional questions about intention --> user receive recommendation based on score<br>2) midwives and social workers: structured telephone interviews<br>3) researcher: Analyzing user behaviour (*Google Analytics* and app access)	1) baseline assessment (sociodemographic questions)<br>2) app download and training<br>3) app use with daily EPDS questions and 2 times (beginning and end) paper based EPDS questions<br>4) 6 weeks postnatal: final interviews + quantitative assessment with MARS score	1) Home visit through researcher team<br>2) Instructions and download application<br>3) App usage: EPDS + sociodemographic data collection<br>4) evaluation of the application<br>5) follow-up recommendation based on their score (normal / mild to moderate / severe depression)
Features of the digital self-assessment tool	• psychoeducational informal video<br>• EPDS self-identifcation<br>• subpage with information on postnatal depression<br>• help subpage with information about further support and treatment options	• daily EPDS Screening via app (accessible for clinic staff)<br>• additional function: randomly daily alert between 9am and 7pm - 2 hours window to answer the questions.<br>• “snooze” function to postpone the alert for half an hour.	• EPDS 12 item Indonesian version<br>• results and recommendation
Stage of self-identification / feedback	self- identification (1)<br>feedback: based on risk status- users receive result and recommendations.	self- identification (3): alert reminds to daily answer the questions.<br>no feedback to user.	self- identification (2). explanations before process.<br>feedback and recommendation based on score (normal / mild to moderate / severe depression)
Information about further help after self-assessment.	Yes, according the score + help subpage	no information is provided.	Yes, recommendations according the score.
Emergency Information	Yes, emergency recommendation when high risk for suicide.	no direct information. Risk of self-harm: response sent to general practitioner within one working day.	n.a.
Recommended pathway	n.a.	n.a.	Yes, recommendation pathway based on EPDS score.
Involvement of professionals	health professionals for recruiting process	the responses could be seen by clinicians / research team via a password- protected web interface.	not involved.
**Comparator**	none	as a part of the study about validity: paper-based version of EPDS	none
**Outcome** parameters analyzed	• user experience<br>• user behaviour<br>• healthcare providers' experience	• feasibility<br>• patterns of app use<br>• validity<br>• safety<br>• acceptability & usability<br>• user experiences (presented in a follow-up paper)	"effectiveness":<br>acceptability and usability
perinatal mental illness type	Postnatal depression	postnatal depression	postnatal depression
Tools for assessing mental health state	EPDS (cutoff ≥ 12)	EPDS (cutoff ≥ 12)	EPDS Indonesian version (12 items, cutoff ≥ 12)
Additional tools used for the study	CSQ-I, SUS, Google Analytics	MARS	own questions- without origin

Authors, Year (citation)	Reilly & Austin (2021)	Highet *et al.* (2019)
**Study intervention**	1) download of *mummatters* app<br>2) answer demographic questions + baseline assessment (*Whooley questions,* ANRQ + postnatal equivalent) --> users receive recommendation<br>3) additional web-based Key Survey: questions relating to the acceptability, credibility, likeability, perceived effect, and motivational appeal of the tool, questions about help-seeking behaviors in the previous month and barriers to help seeking.	1) brief training session with Maternal Child Health nurses<br>2) While waiting for their postnatal check-up, women completed the questions on the *iCope* platform on a digital screen in the waiting room.<br>If a woman needed help, it could be completed during the consultation.<br>3) results: automatically calculated --> report was sent to the clinician, as well as a short report to the participant via mail or text message.
Features of the digital self-assessment tool	• *Whooley questions* & ANQR self-identification<br>• monthly prompts<br>• key feature: computer-based decision aid that combines responses to *the Whooley questions* and ANRQ--> recommendations and<br>help-seeking information.<br>• possibility to create an individualized wellness action plan and option to receive inspirational messages	• EPDS<br>• questions to detect the presence if psychosocial risk<br>• additional questions if mental health and/ or drug and alcohol risk factors exist.
Stage of self-identification / feedback	self- identification (1).<br>feedback and recommendation based on scores.	self- identification (3): answering questions in the clinic waiting room.<br>possibility to receive a result report.
Information about further help after self-assessment.	Yes, help seeking information. With permission the health care provider can be informed about results.	Yes, links for further information and referral pathways to local specialist/ perinatal mental health support services available (Centre of Perinatal Excellence, 2024).
Emergency Information	n.a.	n.a.
Recommended pathway	n.a.	Yes, pathways are available (Diginostic Pty Ltd, 2024)
Involvement of professionals	A letter can be addressed to the health care provider, if women give their permission, directly from the tool with the results.	*iCOPE* is available to all medical professionals in Australia (Diginostic Pty Ltd, 2024). Clinicians get the results report.
**Comparator**	none	none
**Outcome** parameters analyzed	• sociodemographic and psychosocial data (*Whooley questions* + ANQR)<br>• user experience (acceptability, credibility, perceived effect, motivational appeal, likeability)<br>• help-seeking behaviors and barriers	• performance of iCOPE: user engagement<br>• rates of depression and anxiety (EPDS)<br>• psychosocial risk: 13 risk factors were asked.<br>• request for help
perinatal mental illness type	perinatal depression and psychsocial risk	postnatal mental health risk and psychosocial risk
Tools for assessing mental health state	*Whooley questions* and ANQR	EPDS (9-12 moderate, > 12 very high score)
Additional tools used for the study	modified questions from previous studies	own questions

Results of the studies included for secondary outcomes

Authors, Year (citation)	Daehn *et al.* (2023)	Eisner *et al.* (2022)	Lawson *et al.* (2019)
**SOCIAL DOMAIN**			
**Acceptance and usability**			
Acceptance for technology	CSQ score: 8 questions with a average points of 3.86-4.15 each question. 1-5 stars ranking: mean 4.36 (SD 0.77) range 2-5. asked app reuse: 4.07 (SD 0.75) range 2-5 Recommendation to friends: 4.15 (SD 0.8) range 2-5 There was significant more satisfaction in women, who already had a history of postnatal depression.	The results indicate a high overall acceptability / usability (MARS mean score 4.1, SD 0.5). For some participants the app was not that interesting (MARS score 3.3). recommendation: MARS score 4.0. qualitative: useful and acceptable. Users without symptoms found it less useful.	recommendation to women after delivery: 78 %. 87 % preferred screening by text message to other methods.
Usability of the tool	total SUS score (n = 129) was 75.20 and showed good usability.	The app was easy to use for most participants (mean MARS score 4.7). Some participants noted that the test window of 2 hours was too short.	n.r.
User engagement	n = 1874 unique users had 2752 sessions, with 17.9 % returning users. bounce rate was low: 27.1 % average session duration 02:32minutes most visited: landing page (29.3 %) and survey page (15.3 %) most time spent on: help page (01:07 minutes) in contrast to mean time (00:42 minutes)	An average of 67 % completed the daily assessment in the app. 91 % of participants continued to use it for the full study period. Women with a history of depression used it less often. The app engagement with daily EPDS was gradually less over the study duration from beginning to the end.	930 (99 %) women responded at least one of the six screenings, 67 % completed all six screenings. The participation rate declined with each screening with approximately 2 %. Most positive results were detected two weeks after birth with the first screening (49 %).
Experience of mothers / fathers (-to-be)	n.a.	n.a. --> user experiences will be published in a follow-up paper.	n.r.
Experience of health experts	13 health experts rated the app as a good and supportive tool for adressing postnatal depression- both rated with 4.1 (1-5). 12 out of 13 professionals stated that SmartMums is a useful offer.	n.r.	n.r.
**Availability and access**			
Types of technologies available	not specified in paper. homepage: *SmartMoms*- usage via webpage (SmartMoms, 2024)	*Clin Touch DAWN-P* : app stores for *iPhone* and *Android* during the study period.	only for study period: text messages, e-mail, telephone calls
Accessibility	access global	only for patients of Manchester University NHS Foundation Trust and their partners.	only for women, who deliver Mount Sinai Hospital in Toronto and were part of the study
Target groups	mothers in the postnatal period within 12 months.	pregnant women ≥ 36 weeks pregnant and their partners.	mothers in postnatal period within 12 weeks after birth
Authorization stage	n.a.	*"The app did not meet requirements for registration as a medical device by the Medicines and Healthcare products Regulatory Agency (MHRA)"* (Eisner et al., 2022, p 3)	n.a.
Costs	no costs (SmartMoms, 2024). compensation for expenses (drugstore voucher 25-75 €)	no costs. Participants received up to 60£. Smartphones were also available to rent for free.	no costs. Women got a $10 CAD gift card
**Ethical issues**			
Benefits and harms for patients and professionals	n.r.	n.r.	n.r.
Affects on autonomy for patients	n.r.	n.r.	n.r.
Sphere of privacy	n.r.	n.r.	n.r.
Affect on distribution of health care resources	n.r.	n.r.	n.r.
Equity for usage	n.r.	n.r.	n.r.
**ORGANIZATIONAL DOMAIN**			
Organizational consequences	n.r.	n.r.	n.r.
Affect on current work process	n.r.	n.r.	n.r.
Implementation in health system	n.r.	n.r.	n.r.
Management problems	n.r.	n.r.	n.r.
Further pathways	n.r.	n.r.	n.r.
**LEGAL DOMAIN**			
Regulation for permission of digital technologies	n.r.	n.r.	n.r.
Laws / binding rules concerning safety, marketing, licencing, acquisition, usage	n.r.	n.r.	n.r.

Authors, Year (citation)	Nurbaeti *et al.* (2021)	Reilly & Austin (2021)	Highet *et al.* (2019)
**SOCIAL DOMAIN**			
**Acceptance and usability**			
Acceptance for technology	previously used a similar app: 9.17 %<br>recommendation rate: 97.25 %	acceptance: 94-98.6 % felt comfortable in answering the questions.<br>90-91 % would use the app again.<br>78-85 % would tell friends about it.	n.r.
Usability of the tool	download: 55.96 % very easy, 33.94 % easy.<br>fill out procedure: easy for 94.50 %.<br>easy to understand language: very understandable 61.47 %, understandable 34.86 %	expectations met: 80-81 %<br>easy to find information: 88-90 %	n.r.
User engagement	n.r.	n.a.	overall completion screening rate: 99.3 %.<br>144 screens were performed.<br>average time to answer the question was 6.7minutes (SD 3.78).<br>Two women discussed issues related to the screening tools.<br>84 % wanted to receive the results.
Experience of mothers / fathers (-to-be)	beneficial/useful output: 96.33 %<br>reflected the psychological condition: 90.83 %	Most participants regarded the tools as credible (93.2-97.3 %), appealing (78.1-91 %) and potentially helpful (78.1-92.5 %)	n.a.
Experience of health experts	n.r.	n.r.	n.r.
**Availability and access**			
Types of technologies available	app *tes depresi* available in *Google PlayStore*	The web-based tool *Mummatters* is available for free at *Bupa Website* .	web-based *iCOPE* platform is available in health care facilities in Australia.
Accessibility	only available for android- users and in Indonesian language.	not specified. study inclusion: living in Australia.	global acces and availability in multiple languages.
Target groups	Indonesian women in postnatal period.	pregnant women or women in postnatal period	women postnatal (4-6 weeks after birth)
Authorization stage	n.a.	n.a.	not specified. *iCOPE* data security meets all legislative regulatory frameworks for health-related data in Australia.
Costs	n.a.	no costs.	no costs/ health care facilities: implementation of *iCOPE* in the public sector is funded by the Federal Government until 2025 (Diginostic Pty Ltd, 2024)
**Ethical issues**			
Benefits and harms for patients and professionals	n.r.	n.r.	n.r.
Affects on autonomy for patients	n.r.	n.r.	n.r.
Sphere of privacy	n.r.	n.r.	n.r.
Affect on distribution of health care resources	n.r.	n.r.	n.r.
Equity for usage	n.r.	n.r.	n.r.
**ORGANIZATIONAL DOMAIN**			
Organizational consequences	n.r.	n.r.	n.r.
Affect on current work process	n.r.	n.r.	n.r.
Implementation in health system	n.r.	n.r.	currently ongoing
Management problems	n.r.	n.r.	n.r.
Further pathways	n.r.	n.r.	n.r.
**LEGAL DOMAIN**			
Regulation for permission of digital technologies	n.r.	n.r.	n.r.
Laws / binding rules concerning safety, marketing, licencing, acquisition, usage	n.r.	n.r.	n.r.

Review included according to secondary outcome ethical domain

**Authors, Year (citation)**	Fonseca *et al*. (2024)
**objective of the paper**	In this article, the authors discuss the current literature on digital health technologies for the prevention and treatment of perinatal mental disorders. Different types of DHT are described, benefits are highlighted, negative aspects were discussed, and future relevant research topics are mentioned.
**Paper design**	Scientific review article
Funding:	no specific grant. One author was supported by a doctoral grant.
Conflict of interest:	None.
Target population	Women in perinatal context.
Contents	• Different types of e-mental health tools<br>• Benefits<br>• considering aspects<br>• Ethical aspects<br>• Future research issues
Issues researched regarding the review	Ethical aspects.
organizational impact	n.r.
legal issues	n.r.
ethical issues	Ethical standards and regulations are necessary.<br>• Privacy, confidentiality, and data security.<br>• Data storage and transmission (type and how)<br>• informed consent<br>Lots of digital health tools are already available, but they are lacking evidence for efficacy and safety.<br>Anonymity might be an ethical concern.<br>Information, benefits, and risks need to be communicated clearly.<br>Maintaining equality and equal rights

Supplementary Table S2. Table of excluded studies after full text screening.

No	Author	Title	Exclusion reason
1	("The SUPPORT Study: effectiveness and Usability of a Web-Enabled Resource for Postpartum Mental Health," 2022)	The SUPPORT Study: effectiveness and Usability of a Web-Enabled Resource for Postpartum Mental Health	Study design reason: study protocol
2	("Digital assessment of wellbeing in new parents," 2023)	Digital assessment of wellbeing in new parents	Study design reason: study protocol
3	(Camoni *et al*., 2020)	A screening and treatment programme to deal with perinatal anxiety and depression during the COVID-19 pandemic	Others: only available in Italian language
4	(Chrzan-Detkos & Walczak-Kozlowska, 2022)	Postpartum depression crisis since the second lockdown and 'screening paradox': many women identified, very few treated	Outcome reason: Incidence of depression during COVID-19 pandemic
5	(Clarke *et al*., 2024)	Digital screening for mental health in pregnancy and postpartum: A systematic review	Intervention reason: different types of interventions compared
6	(Doherty *et al*., 2018)	A Mobile App for the Self-Report of Psychological Well-Being During Pregnancy (BrightSelf): Qualitative Design Study	Outcome reason: design of mobile technologies
7	(Doherty *et al*., 2019)	Engagement with Mental Health Screening on Mobile Devices: Results from an Antenatal Feasibility Study	Comparison reason: comparing EPDS with EPDS + Ecological Momentary Assessment (EMA) group
8	(Dosani *et al*., 2020)	mHealth and Perinatal Depression in Low-and Middle-Income Countries: A Scoping Review of the Literature	Outcome reason: different interventions
9	(Fijean *et al*., 2024)	Universal screening of postpartum depression with Edinburgh Postpartum Depression Scale: A prospective observational study	Intervention reason: not self-identification

10	(Guille, Henrich, *et al.*, 2024)	Improving the Management of Maternal Mental Health with Digital Health Care	Intervention reason: not screening/ self-identification
11	(Guille, King, *et al.*, 2024)	Text And Telephone Screening And Referral Improved Detection And Treatment Of Maternal Mental Health Conditions	Intervention reason: not self-identification
12	(Hussain-Shamsy *et al.*, 2020)	Mobile health for perinatal depression and anxiety: Scoping review	Intervention reason: different interventions
13	(Inkster *et al.*, 2023)	Understanding the impact of an AI-enabled conversational agent mobile app on users' mental health and wellbeing with a self-reported maternal event: a mixed methods real-world data mHealth study	Intervention reason: includes education and therapy intervention
14	(Jhawar *et al.*, 2022)	Maternal depression: Technology enabled self screening in real time	Intervention reason: validation and development
15	(Kingston, Austin, *et al.*, 2017)	Pregnant women's views on the feasibility and acceptability of web-based mental health e-screening versus paper-based screening: A randomized controlled trial	Intervention reason: not self-identification (tablet vs. paper)
16	(Kingston, Biringer, *et al.*, 2017)	Pregnant Women's Perceptions of the Risks and Benefits of Disclosure During Web-Based Mental Health E-Screening Versus Paper-Based Screening: Randomized Controlled Trial	Intervention reason: not self-identification (tablet vs. paper)
17	(Kingston *et al.*, 2015)	Preferences for mental health screening among pregnant women: A cross-sectional study	Intervention reason: not self-identification (tablet vs. paper)
18	(Learman, 2018)	Screening for Depression in Pregnancy and the Postpartum Period	Study design reason: no scientific study
19	(Li *et al.*, 2020)	Assessing the quality of mobile applications targeting postpartum depression in China	Intervention reason: different interventions

20	(Lucas *et al*., 2023)	Mobile Assessments to Improve Screening and Novel Patient Engagement to Diagnose and Manage Maternal Mental Health (MIND) Study Midpoint Analysis: compliance Rate of Antenatal Screening	Study design reason: abstract only
21	(Marcano-Belisario *et al*., 2017)	Implementation of depression screening in antenatal clinics through tablet computers: results of a feasibility study	Outcome reason: survey layout
22	(Martínez-Borba *et al*., 2018)	The Use of Information and Communication Technologies in Perinatal Depression Screening: A Systematic Review	Intervention reason: different intervention, not self-identification
23	(Martin-Key *et al*., 2021)	Proof-of-Concept Support for the Development and Implementation of a Digital Assessment for Perinatal Mental Health: Mixed Methods Study	Participant reason: pregnancy planned, gave birth in last two years + intervention reason: not self-identification
24	(Nguyen *et al*., 2023)	Self-care interventions for preconception, antenatal, intrapartum and postpartum care: a scoping review	Intervention reason: not self-identification (self- care intervention)
25	(Oğur *et al*., 2023)	Development of a mobile monitoring program for anxiety and depression in pregnancy and evaluation of 3-month results	Intervention reason: not self-identification (online vs. paper)
26	(Osma *et al*., 2014)	Proposal of use of smartphones to evaluate and diagnose depression and anxiety symptoms during pregnancy and after birth	Intervention reason: different interventions (including therapy)
27	(Pineros-Leano *et al*., 2015)	Clinic staff attitudes towards the use of mHealth technology to conduct perinatal depression screenings: A qualitative study	Outcome reason: app development + participant reason: health care professionals
28	(Reilly *et al*., 2020)	A narrative review of studies addressing the clinical effectiveness of perinatal depression screening programs	Intervention reason: no digital health technology

29	(Reilly *et al*., 2019)	A comparison of the interviewer-administered phone and self-complete online versions of the computerized eMINI 6.0 in a sample of pregnant women	Intervention reason: interview questions vs. telephone
30	(Spadaro *et al*., 2022)	mHealth solutions for perinatal mental health: Scoping review and appraisal following the mHealth index and navigation database framework	Intervention reason: different interventions
31	(Stone & Hirshberg, 2024)	Telemedicine and Digital Health Solutions in Intrapartum and Postpartum Care	Study design reason: no scientific study + intervention reason: apps not for mental health
32	(Vanderkruik *et al*., 2021)	Perinatal depression screening using smartphone technology: Exploring uptake, engagement and future directions for the MGH Perinatal Depression Scale (MGHPDS)	Outcome reason: prevalence
33	(Vani *et al*., 2023)	Piloting a prenatal care smartphone application and care navigation intervention at a federally qualified health center	Outcome reason: implementation, prevalence and sociodemographic data
34	(Varma *et al*., 2023)	Acceptability of an mHealth App for Monitoring Perinatal and Postpartum Mental Health: Qualitative Study With Women and Providers	Intervention reason: not screening/ self-identification
35	(Zingg *et al*., 2020)	Digilego for Peripartum Depression: A Novel Patient-Facing Digital Health Instantiation	Intervention reason: app development
	**Handsearch**		
36	(Feldman *et al*., 2021)	A systematic review of mHealth application interventions for peripartum mood disorders: trends and evidence in academia and industry.	Intervention reason: different interventions
37	(Naslund *et al*., 2017)	Digital technology for treating and preventing mental disorders in low-income and middle-income	Intervention reason: different interventions (prevention and therapy included)

		countries: a narrative review of the literature.	
38	(RANZCOG Women's Health Committee, 2021)	Category: Best Practice Statement. Mental Health Care in the Perinatal Period	Intervention reason: not digital technologies
39	(World Health Organization, 2022a)	Guide for integration of perinatal mental health in maternal and child health services	Intervention reason: not digital technologies
